# Amarogentin from *Gentiana rigescens* Franch Exhibits Antiaging and Neuroprotective Effects through Antioxidative Stress

**DOI:** 10.1155/2020/3184019

**Published:** 2020-08-01

**Authors:** Dejene Disasa, Lihong Cheng, Majid Manzoor, Qian Liu, Ying Wang, Lan Xiang, Jianhua Qi

**Affiliations:** College of Pharmaceutical Science, Zhejiang University, 866 Yu Hang Road, Hangzhou, China

## Abstract

In the present study, the replicative lifespan assay of yeast was used to guide the isolation of antiaging substance from *Gentiana rigescens* Franch, a traditional Chinese medicine. A compound with antiaging effect was isolated, and the chemical structure of this molecule as amarogentin was identified by spectral analysis and compared with the reported data. It significantly extended the replicative lifespan of K6001 yeast at doses of 1, 3, and 10 *μ*M. Furthermore, amarogentin improved the survival rate of yeast under oxidative stress by increasing the activities of catalase (CAT), superoxide dismutase (SOD), and glutathione peroxidase (GPx), and these enzymes' gene expression. In addition, this compound did not extend the replicative lifespan of *sod1*, *sod2*, *uth1*, and *skn7* mutants with K6001 background. These results suggested that amarogentin exhibited antiaging effect on yeast via increase of *SOD2*, *CAT*, *GPx* gene expression, enzyme activity, and antioxidative stress. Moreover, we evaluated antioxidant activity of this natural products using PC12 cell system, a useful model for studying the nervous system at the cellular level. Amarogentin significantly improved the survival rate of PC12 cells under H_2_O_2_-induced oxidative stress and increased the activities of SOD and SOD2, and gene expression of *SOD2*, *CAT*, *GPx*, *Nrf2*, and *Bcl-x1*. Meanwhile, the levels of reactive oxygen species (ROS) and malondialdehyde (MDA) of PC12 cells were significantly reduced after treatment of the amarogentin. These results indicated that antioxidative stress play an important role for antiaging and neuroprotection of amarogentin. Interestingly, amarogentin exhibited neuritogenic activity in PC12 cells. Therefore, the natural products, amarogentin from *G. rigescens* with antioxidant activity could be a good candidate molecule to develop drug for treating neurodegenerative diseases.

## 1. Introduction

The number of aging population is rapidly increasing globally [1]. Aging, which is a functional and structural deterioration of cells, tissues, and organs, has been implicated as a risk factor for age-related diseases, such as neurodegenerative disease, cancer, and metabolic diseases [2–5]. Alzheimer's disease (AD) which is the most prevalent neurodegenerative disorder threatens the health of an enormous number of aging populations worldwide. This age-related neurodegenerative disease has no effectively curative drug. Therefore, the discovery of drugs that can effectively delay the inevitable aging process and cure AD is highly desirable. Rapamycine, resveratrol (RES), metformin, and curcumin which exhibit antiaging effects were also reported to have significant neuroprotective effects in AD models, indicating a strong link between aging and neurodegenerative diseases [6–10]. Thus, antiaging substances may be developed as promising drugs to cure neurodegenerative diseases.

Oxidative stresses play a vital role in aging and neurodegenerative disorders [3, 5]. Gradual accumulation of ROS causes disruption of macromolecules such as proteins, DNA, and lipids which are implicated for progression of neurodegenerative disease and aging processes [5]. The superfluous level of ROS causes several damaging effects such as reduction of ATP production and mitochondrial dysfunction. An excessive or low level of mitochondrial ATP production affects the normal function of nerve cells and its response to stress during aging and AD development. Mitochondrial permeability transition pore results in hypertrophy of mitochondria and subsequent cell death [11]. Another consequence of oxidative stress is oxidative damage to DNA, which could potentially lead to cellular dysfunction and death [5, 11]. Excessive ROS could be scavenged by antioxidative enzymes such as SOD, CAT, and GPx [5]. SODs are metalloproteinases that catalyze the conversion of ROS into less harmful products to protect cells against oxidative damage [12]. CAT helps cells to survive by breaking down reactive hydrogen peroxide into products such as water and oxygen. It is used as a therapeutic agent for several diseases related to oxidative stress [13]. Cellular GPx system is part of essential constituents in protecting cells against oxidative stress. It detoxifies hydrogen peroxides in cells. It plays an indispensable role to protect cells from oxidative damage exerted by free radicals especially lipid peroxidation [12]. Lipid peroxidation product, MDA, is widely used as a biomarker to examine the level of oxidative damage [14].

The genus *Gentiana*, a major group in the Gentianaceae family, is found in Asia, Europe, and America [15]. Several important molecules, such as iridoids, secoiridoids, essential oils, xanthones and terpenoids, have been isolated from *Gentiana*. Iridoids and secoiridoids are the major constituents from this genus and are regarded to be responsible for a variety of biological activities [15]. *Gentiana rigescens* Franch (Jian Long Dan in Chinese) is a well-known traditional Chinese medicine (TCM) that is widely distributed in Yunnan Province, Southwest of China. *G. rigescens* is used to treat hepatitis, rheumatism, cholecystitis, and inflammation [16]. “Sheng Nong's Herbal Classic”, a classic book on TCM material medica, states that *G*. *rigescens* improves cognition and has antiaging activity. In our previous study, gentisides A–K, which are 11 novel neuritogenic benzoate-type molecules, were isolated from *G. rigescens*; the mixture of gentisides (n-GS) was confirmed to alleviate the impaired memory of the AD model [17–19]. Natural products are important sources of drugs to reduce or prevent oxidative stresses in humans. In our previous studies, 19 molecules were isolated from natural products which were able to extend the replicative lifespan of yeast via antioxidative stress [20–25]. Therefore, searching for effective molecules with antioxidative stress potential should be considered an important strategy to treat aging and neurodegenerative disorders.

Yeast is one of the well-known bioassay models in aging research because of its low cost, genetic tractability, and short generation time. A unique characteristic of K6001 is that only mother cells can produce daughter cells in a glucose medium but not in a galactose medium [26]. In our previous study, K6001, which is a yeast mutant strain, was used as a bioassay system to evaluate the antiaging activity of natural products [20–25]. Furthermore, the PC12 cell line, which was derived from rat pheochromocytoma cells, is one of the useful models for studying the nervous system at the cellular level [27]. Based on the link between aging and neurodegenerative diseases [6–10], PC12 cells were also used to evaluate the neuroprotective activity of antiaging molecules.

In present study, a K6001 yeast bioassay system was employed to guide the isolation of antiaging substances. Amarogentin was discovered as an antiaging natural product. Amarogentin is a kind of secoiridoid-type compound that can be found in *Gentiana lutea*, *Swertia japonica*, *Gentianella nitida*, and *Swertia chirayita* [28–30]. It has been reported to possess various activities, such as anti-inflammatory, immunomodulatory, antioxidative stress, and antidiabetic effects [31–33]. Herein, the antiaging, neuroprotective, and neuritogenic effects and the underlying mechanism of the natural bioactive product, amarogentin, which possesses antioxidative stress potential for treatment neurodegenerative disease, will be reported.

## 2. Materials and Methods

### 2.1. General

Silica gel (200–300 mesh, Yantai Chemical Industry Research Institute, Yantai, China) and reversed phase C18 (Octadecylsilyl, ODS) silica gel (Cosmosil 75 C_18_ OPN, Nacalai Tesque, Japan) were used for column chromatography. Precoated silica gel (0.25 mm) and RP-18 plate (0.25 mm) were used for TLC analysis. Preparative high-performance liquid chromatography (HPLC) was performed using a HPLC equipped with two ELITE P-230 pumps and a UV detector. High-resolution electrospray ionisation mass spectrometry (HR-MS) analysis was performed on Agilent 6224A accurate mass time-of-flight LC/MS system (Agilent Technologies). A Bruker AV III-500 spectrometer (Bruker, Billerica, MA, USA) was operated for NMR measurement. The NMR chemical shifts in *δ* (ppm) were referred to the solvent peak of *δ*_C_ (49.0) for methanol.

### 2.2. Extraction and Isolation

Dried roots of *G. rigescens* were purchased from HuQingYuTang Pharmacy in Hangzhou City, Zhejiang Province, China. The plant material (1.5 kg dry weight) was smashed and extracted with methanol for 48 h under shaking at room temperature. The extract was filtered, and the supernatant was concentrated to obtain 400 g of crude extract. The crude extract was partitioned between the water and ethyl acetate. The samples from each layer were tested for antiaging activity on the K6001 yeast strain. The active ethyl acetate layer (12 g) was subjected to silica open column and eluted with *n*-hexane/ethyl acetate (100/0, 90/10, 80/20, 70/30, 60/40, 50/50, 30/70, and 0/100 and methanol 100%) to obtain nine fractions. The active fraction (300 mg) obtained from *n*-hexane/ethyl acetate (30/70) was further chromatographed on ODS and eluted with methanol/water (30/70, 40/60, 50/50 70/30, 80/20, 90/10, and 100/0) to obtain six fractions. The active fraction obtained from 50/50 methanol/water (19 mg) was subjected to HPLC purification (Develosil 5C18-MS-II (10 × 250 mm), flow rate of 3 ml/min and 40% aqueous methanol) to obtain the active sample (11 mg, *t*_R_ = 25 min). In the present study, amarogentin was dissolved in ethanol or DMSO with stock concentration of 10 mM for yeast and PC12 cell experiments, respectively.

### 2.3. Yeast Strains, Culture Medium, and Lifespan Assay

K6001 yeast with back ground W303, wild-type BY4741 yeast strain, and *uth1*, *skn7*, *sod1*, and *sod2* mutants with K6001 background were used. The liquid culture medium contained yeast extract, peptone, and D-glucose (YPD, 1% yeast extract, 2% peptone and 2% glucose) or galactose (YPG, 1% yeast extract, 2% peptone, 3% galactose) media. Replicative lifespan assays were performed as described in our previous study [20]. In brief, the K6001 yeast strain was inoculated in 5 ml of galactose medium and incubated in a shaking incubator at 180 rpm for 24 h at 28°C. Afterwards, 1 ml of broth containing yeast was centrifuged, and the yeast pellet was washed three times with phosphate buffer solution (PBS). The pellet was then diluted with PBS, and a haemocytometer was used to count cells. Approximately 4,000 cells were smeared on the glucose medium agar plates containing resveratrol (RES) or amarogentin at different concentrations. The agar plates were incubated for 48 h at 28°C, and the microcolonies that formed on the agar plate were observed under a microscope. Forty microcolonies were randomly chosen to count the number of daughter cells produced by one mother cell. The replicative lifespan assay of *uth1*, *skn7*, *sod1*, and *sod2* mutants with K6001 background was the same as that of K6001 yeast strain.

### 2.4. Antioxidative Stress Assay

Based on the evidence from our previous studies [21, 23], 10 mM H_2_O_2_ was chosen as the optimum concentration to induce oxidative stress. The wild-type BY4741 yeast was inoculated in YPD medium, placed in a shaker incubator, and cultured for 24 h. The BY4741 yeast at initial 0.1 OD was placed in a liquid glucose medium and treated with RES at 10 *μ*M as positive control or amarogentin (0, 1, 3 and 10 *μ*M) for 24 h at 28°C. Subsequently, 5 *μ*l of the cultured cells with the same OD600 value from each group was dropped on a plate containing 10 mM H_2_O_2_. The growth of yeast cells on the plate was observed and photographed after 3 days of incubation at 28°C.

The effect of amarogentin on oxidative stress in yeast was quantified using a different approach. Similar to the above antioxidative stress assessment, BY4741 yeast cells were treated with RES (10 *μ*M) or amarogentin (0, 1, 3, and 10 *μ*M). The counted 200 yeast cells from each group were spread on a glucose agar plate with or without 5.5 mM H_2_O_2_ and incubated at 28°C for 48 h. After 2 days, the colonies that formed on the plate were counted. The survival rate of the yeast cells was analysed from the ratio of the number of colonies in the absence of 5.5 mM H_2_O_2_ divided by the number of colonies in the presence of 5.5 mM H_2_O_2_.

### 2.5. Measurement of SOD, GPx, and CAT Enzymatic Activities in Yeast

Y4741 yeast in the liquid glucose medium was treated with RES (10 *μ*M) or amarogentin (0, 1, 3, and 10 *μ*M) for 48 h at 28°C. The activity assays of total SOD, SOD1, SOD2, GPx, and CAT were performed according to a previous study [23, 24]. The BY4741 yeast cells in the liquid glucose medium was treated with RES (10 *μ*M) or amarogentin (0, 1, 3, and 10 *μ*M) for 24 h at 28°C with the initial OD of 0.1. Yeast cells were collected and sonicated for five minutes (each time lasted for 1 min). The cell lysates were centrifuged to get supernatant. The enzymatic activities of the SOD, GPx, and CAT in the supernatant were measured with corresponding assay kits (Biotime Biotechnology Limited Company, Shanghai, China) following the manufacturer's instructions; the details of assays procedures are shown in supplementary materials.

### 2.6. Neuroprotection Effect of Amarogentin in PC12 Cells

Approximately 50,000 PC12 cells were seeded in each well of a 24-well plate and cultured in 5% CO_2_ at 37°C for 24 h. Then, the medium was replaced by 1 ml serum-free Dulbecco's modified Eagle's medium (DMEM; Thermo Scientific, Shanghai, China) containing different tested samples. In the dose-dependent experiment of H_2_O_2_, the cells were treated with 0.5% dimethyl sulphoxide (DMSO) for 24 h and cultured with different concentrations of H_2_O_2_ for 1 h. To investigate the neuroprotection effect of amarogentin, PC12 cells were treated with RES (10 *μ*M) or amarogentin (1, 3, or 10 *μ*M) for 24 h and with 0.9 mM H_2_O_2_ for 1 h. The medium was replaced with 500 *μ*l of serum-free DMEM containing 200 *μ*g/ml 3-(4,5-dimethyltaizol-2-yl)-2,5-diphenyltetrazolium bromide (MTT) and cultured for another 2 h. The medium was completely removed and replaced by 200 *μ*l of DMSO to each well to solubilise the formed formazan crystal. The resultant formazan was detected at 570 nm by using a plate reader (Bio-Tec instruments Inc., Winooski, VT, USA). The experiment was performed three times, and the result was considered viable compared with negative control.

### 2.7. ROS, MDA, Total SOD, SOD1, and SOD2 Enzymatic Activity Assays in PC12 Cells

To determine the ROS level in PC12 cells, approximately 50,000 PC12 cells were seeded in each well of a 24-well plate. The cells were treated with RES (10 *μ*M) or amarogentin (1, 3, and 10 *μ*M) for 24 h and then with 0.9 mM H_2_O_2_ for 1 h. Each well was then added with DCFH-DA (2′,7′-dichlorodihydrofluorescein diacetate, final concentration, 10 *μ*M) and incubated for 30 min. The cells were washed with PBS to remove extracellular DCFH-DA, and intercellular ROS was detected by using the SpectraMax M3 multimode microplate reader (Molecular Devices Corporation, California, USA) under the excitation wavelength of 488 nm and emission wavelength of 525 nm. At the same time, DCF in PC12 cells was observed using a fluorescence microscope (HCS, Thermo Fisher, scientific, Waltham, MA, USA).

To test the MDA content and total SOD, SOD1, and SOD2 enzymatic activities in PC12 cells, approximately 2 × 10^6^ of PC12 cells were seeded in a 60 mm culture dish, containing 5 ml of DMEM medium, and incubated for 24 h. Then, PC12 cells were treated with RES (10 *μ*M) or amarogentin (1, 3, or 10 *μ*M) for 24 h and then with 0.9 mM H_2_O_2_ for another 1 h to determine MDA content, and total SOD, SOD1, and SOD2 activities. The cells were then collected by centrifugation and three cycles of ultrasonication (1 min for each instance) with PBS. The cell lysates were centrifuged, and the supernatant was removed to assess MDA, total SOD, SOD1, and SOD2 enzymatic activities. MDA quantification and total SOD, SOD1, and SOD2 activities were determined using MDA and SOD enzymatic activity assay kits (Nanjing Jiancheng Bioengineering Institute, Nanjing, China) following the manufacturer's instructions; the details of assays procedures are shown in supplementary materials.

### 2.8. RT-PCR Analysis

To get the RNA samples of yeast, BY4741 yeast cells were cultured in glucose medium >following addition of 0, 1, 3, and 10 *μ*M of amarogentin or 10 *μ*M RES. RNA was extracted from yeast cells in the exponential phase through the hot phenol method. For PC12 cells, approximately 2 × 10^6^ of PC12 cells were seeded in a 60 mm culture dish and treated with RES (10 *μ*M) or amarogentin (1, 3, or 10 *μ*M) for 12 h or 24 h. Total RNA was extracted with TRIzol Reagent (Beijing Cowin Biotech Company, Beijing, China). RNA content was determined using Eppendorf Biophotometer Plus (Eppendorf Company, Hamburg, Germany). Transcription was performed using 5 *μ*g of total RNA, Oligo (dT)_20_ primers, and reverse transcriptase (Beijing Cowin Biotech Company, Beijing, China). Transcript levels were quantified by real-time PCR (AB SCIEX, Massachusetts, USA) and SYBR Premix EX Taq™ (Takara, Otsu, Japan). The primers (Sangon Biotech Co. Ltd., Shanghai, China) used in this study are given in Supplementary Table S1. Thermal recycling parameters for yeast are as follows: *SOD1* and *SOD2*, 95°C for 2 min, followed by 40 cycles, 94°C for 15 seconds, 60°C for 25 seconds, and 72°C for 10 seconds; and *GPx* and *CAT*, 40 cycles, 95°C for 15 seconds, 60°C for 35 seconds. Thermal recycling parameters for PC12 cells are as follows: *SOD1* and *SOD2*: 95°C for 2 min, followed by 40 cycles, 54°C for 35 seconds; *GPx* and *CAT*, 95°C for 2 min, followed by 40 cycles, 55°C for 35 seconds; and *Bcl-x1* and *Nrf2*: 95°C for 2 min, followed by 40 cycles, 95°C for 15 seconds, 60°C for 15 seconds, and 68°C for 20 seconds. All results were normalized to *TUB1* or *GAPDH* RNA levels, and relative mRNA transcript levels were calculated using the *ΔΔ*Ct formula. All samples were run in triplicate, and the average values were calculated.

### 2.9. Neuritogenic Activity of Amarogentin in PC12 Cells

The neuritogenic activity of the isolated compound was evaluated according to our previous report [17]. Approximately 50,000 PC12 cells were seeded in each well of a 24-well microplate in 1 ml of DMEM containing 7.5% foetal bovine serum, 10% horse serum, and 1% premixed antibiotics (Invitrogen, Shanghai, China) and cultured under a humidified atmosphere of 5% CO_2_ at 37°C. The medium was replaced by serum-free DMEM containing either DMSO (0.5%) or the sample at various concentrations after 24 h. The cells were treated with 40 ng/ml NGF (Recombinant Human *β*-NGF, Sigma, Shanghai, China) as the positive control. For evaluation of the enhancement of NGF activity, the medium was replaced by serum-free DMEM containing 1 ng/ml NGF in addition to the test sample. Cellular morphological changes were observed under a phase-contrast microscope (Olympus, model CKX41, Tokyo, Japan) after 48 h. Approximately 100 cells were randomly selected from different areas and counted. Cells bearing neuritis outgrowth longer than the diameter of the cell body were considered positive cells.

### 2.10. Statistical Analysis

Data were evaluated by one-way ANOVA, followed by Tukey's post hoc test by using GraphPad Prism software. *p* value < 0.05 was considered statistically significant. Each experiment was repeated three times, and data were expressed as mean ± SEM.

## 3. Results

### 3.1. Extraction and Isolation

**Figure 1:**
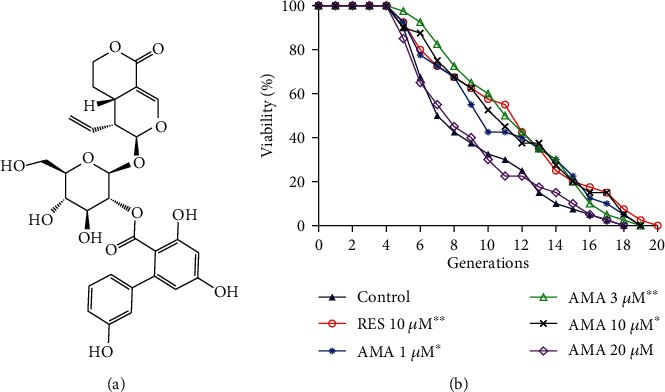
Chemical structure of amarogentin (AMA) (a), changes in the replicative lifespan of K6001 yeast after treatment with different concentrations of amarogentin (b). In the replicative lifespan assay, K6001 yeast cultured in galactose medium for 24 h. After that, it was spread on glucose agar plates with different concentrations of amarogentin or 10 *μ*M RES as a positive control and incubated for 48 h. The randomly selected daughter cells produced by 40 mother cells were counted, and the survival curve was plotted for analysis. This process was repeated three times. ^∗^, ^∗∗^ indicated significant difference as compared with the control (*p* < 0.05 and 0.01), respectively.

The methanol extract of *G. rigescens* was partitioned between ethyl acetate and water to obtain two samples from each layer, respectively. The two samples were tested on the K6001 yeast strain to evaluate their antiaging activity. The active ethyl acetate layer sample was subjected to silica gel and then ODS open column followed by HPLC purification to yield the active molecule (11 mg). The chemical structure of the molecule was identified as amarogentin (AMA) (Figure 1(a)) by comparing spectral data with those reported in literature [34]. ^13^C NMR (125 MHz, methanol-*d_4_*): *δ* 25.8, 28.7, 43.4, 62.4, 69.5, 71.7, 74.6, 74.8, 78.4, 96.8, 97.2, 103.1, 104.0, 105.6, 112.9, 114.5, 116.5, 121.0, 121.2, 129.3, 132.9, 146.5, 148.6, 153.7, 157.5, 163.9, 166.0, 167.6 and 171.5; HRESI-TOF-MS, *m*/*z* 609.1584, calculated for C_29_H_30_O_13_Na (M+Na)^+^ 609.1579.

### 3.2. Amarogentin Extended the Replicative Lifespan of Yeast with K6001 Background

The K6001 yeast strain has a unique characteristic; that is, daughter cells produced by mother cells only survive in a galactose medium and not in a glucose medium [23]. Therefore, we utilized this characteristic to perform a replicative lifespan assay. The effect of amarogentin on the replicative lifespan of the K6001 yeast strain is displayed in Figure 1(b). The mean lifespan of the control is 7.8 ± 0.1 generations, 10.4 ± 0.1 in the RES-treated group, and 9.8 ± 2.4, 10.6 ± 2.0, 9.8 ± 0.1, and 8.1 ± 0.2 generations in amarogentin groups at concentrations of 1, 3, 10, and 20 *μ*M, respectively. Amarogentin extended the replicative lifespan of K6001 yeast at concentrations of 1, 3, and 10 *μ*M compared with the control group (*p* < 0.05, *p* < 0.01, and *p* < 0.05, respectively). Hence, 3 *μ*M amarogentin is the best concentration. The result also suggests that amarogentin has antiaging effect on yeasts.

### 3.3. Amarogentin Improved the Survival Rate of Yeast under Oxidative Stress and SOD, GPx, and CAT Activities

**Figure 2:**
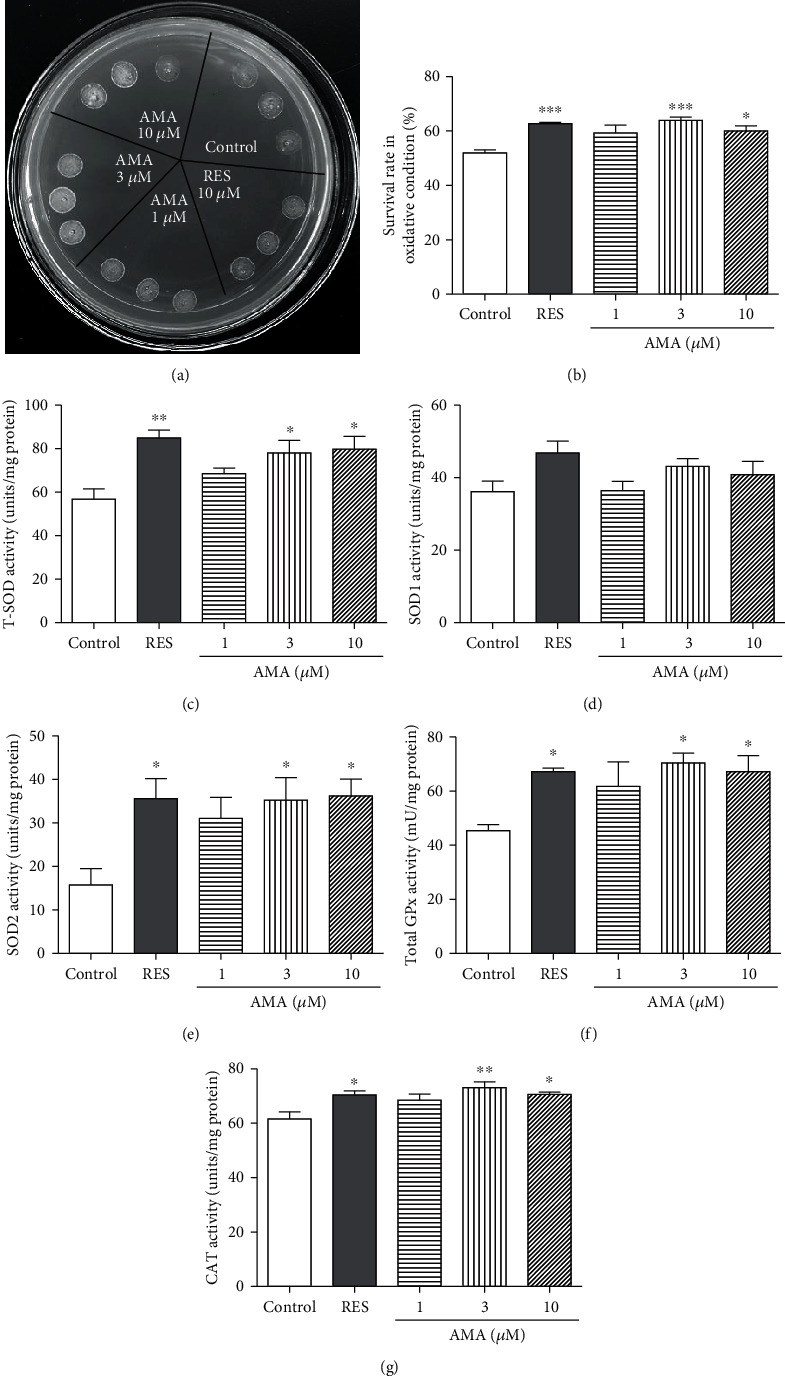
Effect of amarogentin on oxidative stress in yeast and the SOD, GPx, and CAT enzymatic activities of yeast. (a) The growth of yeast under oxidative stress induced by 10 mM H_2_O_2_ after treatment with amarogentin. The wild-type BY4741 yeast was inoculated in YPD medium for 24 h. Then after, initial 0.1 OD of yeast was placed in a liquid glucose medium and treated with RES at 10 *μ*M as positive control or amarogentin (0, 1, 3, and 10 *μ*M) for 24 h at 28°C. Subsequently, 5 *μ*l of the cultured cells with the same OD600 value from each group was dropped on a plate containing 10 mM H_2_O_2_. The growth of yeast cells on the plate was observed and photographed after 3 days of incubation at 28°C. (b) Survival rate of the BY4741 yeast strain under 5.5 mM H_2_O_2_-induced yeast oxidative stress after adding amarogentin. BY4741 yeast cells were treated with RES (10 *μ*M) or amarogentin (0, 1, 3, and 10 *μ*M). The counted 200 yeast cells from each group were spread on a glucose agar plate with or without 5.5 mM H_2_O_2_ and incubated at 28°C for 48 h. After 2 days, the colonies that formed on the plate were counted. The survival rate of yeast cells was analysed from the ratio of the number of colonies in the absence of 5.5 mM H_2_O_2_ divided by the number of colonies in the presence of 5.5 mM H_2_O_2_. Effect of amarogentin on the total superoxide dismutase (T-SOD) (c), SOD1 (d), SOD2 (e), total glutathione peroxidase (GPx) (f) and catalase (CAT) (g). BY4741 yeast strain cells were incubated with RES as a positive control or amarogentin at concentrations of 1, 3, and 10 *μ*M for 24 h. Afterwards, the yeast cells were collected by centrifugation and ultrasonicated for five times. The supernatant of the yeast was used to measure the T-SOD, SOD1, SOD2, GPx, and CAT activities according to the manufacturer's instructions. Each experiment was conducted thrice. ^∗^, ^∗∗^, and ^∗∗∗^ indicated significant difference as compared with the control (*p* < 0.05, 0.01, and 0.001), respectively.

Oxidative stress is one of the major risk factors for aging and age-related pathologies. The elevated level of oxidants or free radicals released from biochemical reactions triggers deleterious effect on macromolecules, such as proteins, nucleic acids, and lipids [11, 12]. Therefore, we focused on the antioxidative stress to do investigation with two methods. The effects of amarogentin on the growth of yeast under oxidative stress induced by 10 mM H_2_O_2_ are shown in Figure 2(a). The growth of yeast on the agar plate with H_2_O_2_ in the amarogentin-treated groups was better than negative control and RES-treated groups. To quantify the change induced by amarogentin on oxidative stress, we used another analytical method. The survival rate of yeast under oxidative stress induced by 5.5 mM H_2_O_2_ in the amarogentin-treated group was significantly increased compared with that in the control group (Figure 2(b)). The effect of amarogentin on antioxidative stress at a concentration of 3 *μ*M is similar to that of RES at a concentration of 10 *μ*M. These results indicate that amarogentin showed antiaging effect by inhibiting oxidative stress.

Antioxidant enzyme system widely exists in organisms. It can effectively eliminate the active oxygen produced by the metabolism of organisms and protect the biological macromolecules from the oxidative damage of active oxygen radicals. The antioxidant enzyme system is mainly composed of superoxide dismutase, glutathione peroxidase, and catalase [5]. Thus, we evaluated the activities of total SOD, SOD1, SOD2, CAT, and total GPx in yeast after treatment with amarogentin at different concentrations for 24 h. As indicated in Figures 2(c)–2(g), the enzymatic activities of total SOD, SOD2, CAT, and GPx notably increased after treatment with 3 and 10 *μ*M amarogentin. The SOD1 activity was not affected by amarogentin. Therefore, amarogentin exhibited antiaging effect by regulating the total SOD, SOD2, GPx, and CAT enzymatic activities.

### 3.4. Effect of Amarogentin on Gene Expression of SOD1, SOD2, GPx, and CAT in Yeast

**Figure 3:**
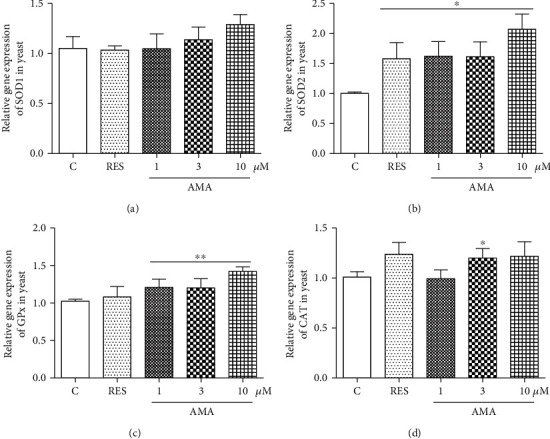
Effect of amarogentin on *SOD1*, *SOD2*, *GPx*, and *CAT* gene expression in BY4741 yeast after treatment of amarogentin and RES. Experiments were repeated thrice, and the data were presented as means ± SEM. ^∗^*p* < 0.05 and ^∗∗^*p* < 0.01 represent significant difference compared with the control group.

The changes on *SOD1*, *SOD2*, *GPx*, and *CAT* gene expression of yeast are shown in Figure 3. The gene expression of *SOD1* was not affected by amarogentin (Figure 3(a)). The abundance of *SOD2* mRNA was significantly increased after treatment of amarogentin at doses of 1 and 3 *μ*M (Figure 3(b), *p* < 0.05, *p* < 0.05). The significant increase of *GPx* gene expression was observed in all of amarogentin-treated groups (Figure 3(c), *p* < 0.01, *p* < 0.01, and *p* < 0.01). The significant increase of *CAT* gene expression was only observed in the 3 *μ*M amarogentin-treated group (Figure 3(d), *p* < 0.05). These results suggested that *SOD2*, *GPx*, and *CAT* genes took important roles in antiaging effect of amarogentin.

### 3.5. Amarogentin Failed to Extend the Replicative Lifespan of sod1, sod2, uth1, and skn7 Mutants of Yeast with K6001 Background

**Figure 4:**
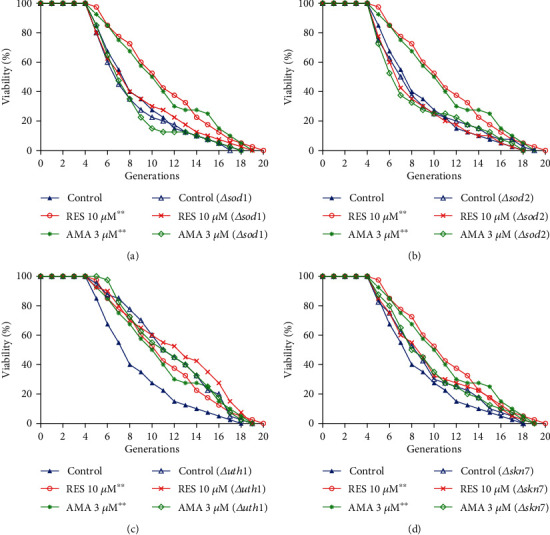
Effect of amarogentin on the replicative lifespans of *sod1* (a), *sod2* (b), *uth1* (c), and *skn7* (d) mutant yeast strain with K6001 background. The average replicative lifespans of the wild-type K6001 yeast were 7.0 ± 0.3 generations under the control treatment, 9.0 ± 0.5 generations under the RES treatment, and 9.6 ± 0.5 generations under the 3 *μ*M amarogentin treatment. The average replicative lifespans of the *sod1* mutant were 7.5 ± 0.1 generations under the control treatment, 7.6 ± 0.3 generations under the RES treatment, and 7.6 ± 0.2 generations under the 3 *μ*M amarogentin treatment. The average replicative lifespans of the *sod2* mutant were 8.1 ± 0.1 generations under the control treatment, 8.1 ± 0.5 generations under the RES treatment, and 7.7 ± 0.2 generations under the 3 *μ*M amarogentin treatment. The average replicative lifespans of the *uth1* mutant were 10.8 ± 0.1 generations under the control treatment, 11.3 ± 0.1 generations under the RES treatment, and 11.0 ± 0.1 generations under the 3 *μ*M amarogentin treatment. The average replicative lifespans of *skn7* were 8.5 ± 0.7 generations under the control treatment, 8.1 ± 0.6 generations under RES treatment, and 8.0 ± 0.6 generations under 3 *μ*M amarogentin treatment.

Several genes are known to be involved in regulating the aging process. *UTH1*, which is an aging gene, is involved in apoptosis. *UTH1* gene intrinsically participates in regulating oxidative stress, and the deletion of this gene leads to extended replicative life span in yeast. *SKN7* is an upstream transcriptional factor for *UTH1*, which is associated in the defence against oxidative stress [35, 36]. SOD is a kind of antioxidant metal enzyme in an organism. This molecule can catalyse the superoxide anion free radical disproportionation to produce oxygen and hydrogen peroxide. SOD plays an important role in the balance of oxidation and antioxidation and is closely related to the occurrence and development of many diseases. To investigate whether these proteins and gene were involved in the anti-aging effect of amarogentin, we used *sod1*, *sod2*, *uth1*, and *skn7* mutants of yeast with K6001 background to perform the replicative lifespan assay. The changes in the replicative lifespan of mutant yeast with K6001 background after treatment with amarogentin are shown in Figures 4(a)–4(d). The replicative lifespan in *Δuth1* yeast significantly increased compared with that in K6001 yeast. However, the replicative lifespans of the mutants were not affected by amarogentin and RES. These results indicate that *SOD1*, *SOD2*, *UTH1*, and *SKN7* genes were involved in the antiaging effect of amarogentin.

### 3.6. Amarogentin Exhibited Neuroprotection on the H_2_O_2_-Induced Oxidative Damage in PC12 Cells

**Figure 5:**
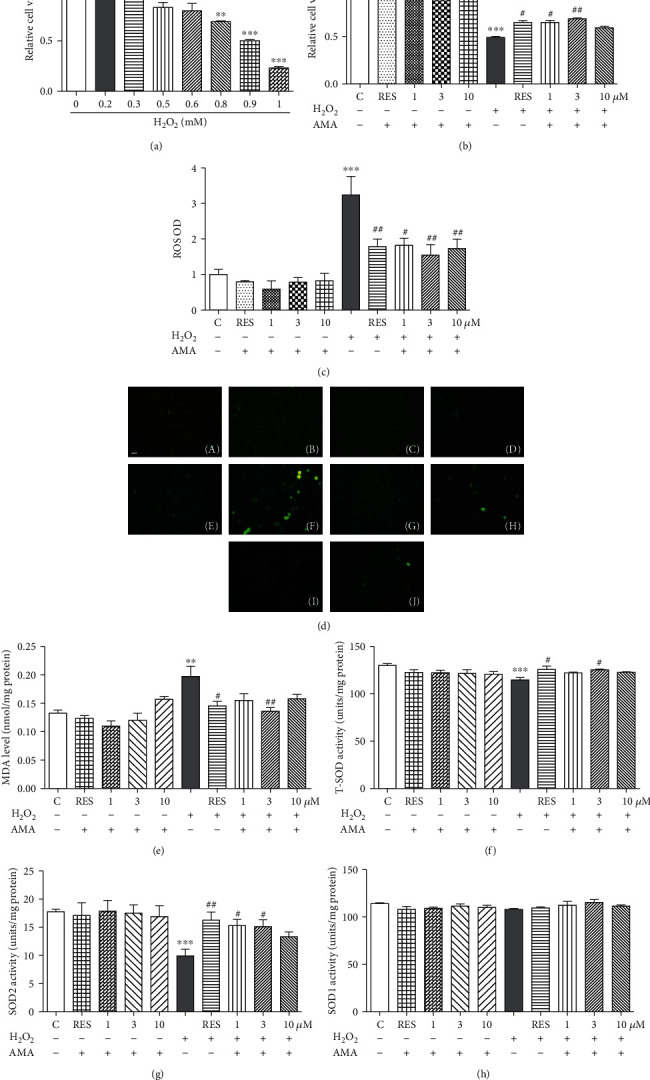
Neuroprotection effect of amarogentin on the H_2_O_2_-induced oxidative damage in the PC12 cells. (a) Relative viability of the PC12 cells after treatment with H_2_O_2_ at different concentrations for 1 h. With an increase in H_2_O_2_ concentration, survival rates decreased significantly compared with the control group. (b) Neuronal protection of amarogentin at 1, 3, and 10 *μ*M with or without the H_2_O_2_ stimulation. (c) Effect of amarogentin with or without H_2_O_2_-induced on ROS production in PC12 cells as detected by fluorescence microplate reader. (d) Photomicrographs of PC12 cells stained with DCFH-DA under a fluorescence microscope. Control (A), RES (10 *μ*M) (B), amarogentin (1 *μ*M) (C), amarogentin (3 *μ*M) (D), amarogentin (10 *μ*M) (E), H_2_O_2_-treated control (F), H_2_O_2_+RES (10 *μ*M) (G), H_2_O_2_+amarogentin (1 *μ*M) (H), H_2_O_2_+amarogentin (3 *μ*M) (I), and H_2_O_2_+amarogentin (10 *μ*M) (J); scale bar, 20 *μ*m. The levels of MDA content (e), total SOD activities (f), SOD2 activities (g), and SOD1 activities (h) were measured by corresponding assay kits. The PC12 cells were pretreated with RES (10 *μ*M) and different concentrations of amarogentin (1, 3, and 10 *μ*M) for 24 h and then subjected to H_2_O_2_ (0.9 mM) for 1 h or treated with RES (10 *μ*M) and amarogentin (1, 3, and 10 *μ*M) alone for 24 h. Experiments were repeated thrice, and the data were presented as means ± SEM. ^∗^*p* < 0.05, ^∗∗^*p* < 0.01, and ^∗∗∗^*p* < 0.001 compared with the control group; ^#^*p* < 0.05, ^##^*p* < 0.01, compared with the H_2_O_2_-treated group (grey color bar).

Antioxidative stress is one of the essential pathways for protecting against neurodegeneration. Based on the significant antioxidative activity of amarogentin in yeast, the PC12 cell line, which was derived from mammalian cells, was employed as a bioassay system to confirm the effects of the antioxidative stress of amarogentin in higher organisms. H_2_O_2_ was used to induce oxidative stress in PC12 cells, and the effect of amarogentin on cell viability was assessed by the MTT method.  Figure 5(a) shows that the cell viability decreased dose-dependently with increasing concentration of H_2_O_2_ from 0.2 mM to 1 mM. This result indicates that approximately 50% of the cells were dead or under poor condition after 1 h of treatment with 0.9 mM H_2_O_2_. According to the cell viability induced by H_2_O_2_, 0.9 mM H_2_O_2_ was chosen as the optimum concentration to induce oxidative stress in PC12 cells. Amarogentin at 1 *μ*M and 3 *μ*M and RES at 10 *μ*M significantly increase survival rates of PC12 cells (Figure 5(b)). These results demonstrate that amarogentin exhibited a neuroprotective effect on PC12 cells.

### 3.7. Amarogentin Decreased Intracellular ROS Level and MDA Content under Oxidative Stress Induced by H_2_O_2_ in PC12 Cells

ROS is one of the main causes of many age-related diseases, such as AD. In addition, ROS is an effector of oxidative stress in cells [37]. Therefore, the protection effect of amarogentin against ROS production induced by H_2_O_2_ was investigated. The intracellular formation of ROS in the PC12 cells was monitored by using the DCFH-DA assay. The nonfluorescent DCFH-DA can be oxidised to a green fluorescent substance (DCF) when it reacts with ROS [38]. As shown in Figure 5(c), the fluorescence intensity was enhanced after treatment with 0.9 mM H_2_O_2_ for 1 h. However, the increasing fluorescence intensity by H_2_O_2_ was diminished after adding 1, 3, and 10 *μ*M amarogentin. The photomicrographs of PC12 cells under fluorescence microscope are shown in Figure 5(d). These results suggest that amarogentin is essential to counteract an oxidative insult to cells rendered by the highly reactive ROS induced by H_2_O_2_.

Lipids are among the classes of biological macromolecules that are targeted by oxidative substances. Lipid oxidation results in the formation of numerous metabolites, which are mainly aldehydes. Metabolites from lipid peroxidation can interact with other macromolecules, such as nucleic acids and protein; such interaction most often results in irreversible damage to cellular function. MDA, which is the principal and most studied product of polyunsaturated fatty acid peroxidation, is often considered a biomarker of oxidative stress [14]. Therefore, the MDA level in H_2_O_2_-induced oxidative damage to PC12 cells was evaluated. The MDA level increased significantly in the H_2_O_2_-treated cells compared with the untreated control group. However, the MDA level in the amarogentin-treated group was significantly decreased compared with that in the H_2_O_2_-treated group. This result confirms that the pretreatment with amarogentin inhibited lipid peroxidation, reduced the level of MDA formation, and rescued cells from damage (Figure 5(e)).

### 3.8. Amarogentin Increased the Total SOD and SOD2 Activity under Oxidative Stress Induced by H_2_O_2_ in PC12 Cells

SOD is an important endogenous free radical scavenger in mammalian cells [39]. Therefore, the level of SOD in the H_2_O_2_-induced oxidative damage to PC12 cells was evaluated. The total SOD and SOD2 activities were significantly decreased in the H_2_O_2_-treated group. The total SOD and SOD2 activities evidently increased in amarogentin-treated cells (Figures 5(f) and 5(g)). However, SOD1 activity in PC12 cells was not affected by amarogentin (Figure 5(h)). Furthermore, amarogentin and RES alone did not affect the cell viability, fluorescence intensity, MDA level, and the total SOD, SOD1, and SOD2 activities in normal condition. These results indicate that amarogentin could significantly reduce the oxidative damage in PC12 cells.

### 3.9. Effect of Amarogentin on Gene Expression of SOD1, SOD2, Bcl-x1, and Nrf2 in PC12 Cells

**Figure 6:**
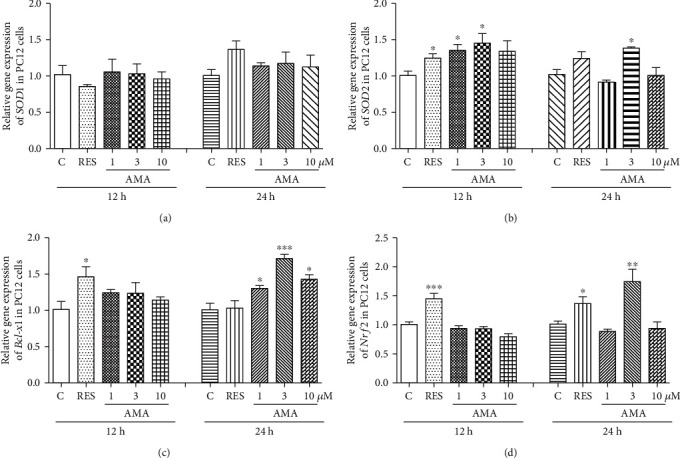
Effect of amarogentin on *SOD1*, *SOD2*, *Bcl-x1*, and *Nrf2* gene expression in PC12 cells after treatment of amarogentin and resveratrol for 12 h or 24 h. Experiments were repeated thrice, and the data were presented as mean ± SEM. ^∗^*p* < 0.05, ^∗∗^*p* < 0.01, and ^∗∗∗^*p* < 0.001 compared with the control group.

The gene expressions of *SOD1* and *SOD2* in PC12 cells after treatment of RES or amarogentin are shown in Figure 6. The *SOD1* gene expression in all of treatment groups was not affected by RES or amarogentin (Figure 6(a)). However, *SOD2* gene expression in these groups was significantly increased after treatment with RES (10 *μ*M) or amarogentin (1, 3 *μ*M) for 12 h (Figure 6(b)). In addition, we detected the gene expression of *GPx* and *CAT*. The abundance of *GPx* and *CAT* mRNA was also significantly increased with RES (10 *μ*M) or amarogentin (1, 3 *μ*M) for 12 h or 24 h (Supplementary Figure 1).

Nrf2 plays an important role in protecting against oxidative stress and apoptotic damage [40]. Bcl-xl, one of the antiapoptotic proteins, plays a considerable role of resistance in apoptosis and involves neuroprotection [41]. Thus, we conducted qRT-PCR to detect whether *Nrf2* and *Bcl-x1* genes were involved in the protection effect of amarogentin. The abundance of *Bcl-xl* and *Nrf2* mRNA was significantly increased with RES or amarogentin (1, 3, and 10 *μ*M) for 12 h or 24 h (Figures 6(c) and 6(d)). These results indicated that *SOD2*, *CAT*, *Gpx*, *Bcl-x1*, and *Nrf2* genes were involved in the neuroprotective effects of amarogentin.

### 3.10. Amarogentin Showed Neuritogenic Activity in PC12 Cells

**Figure 7:**
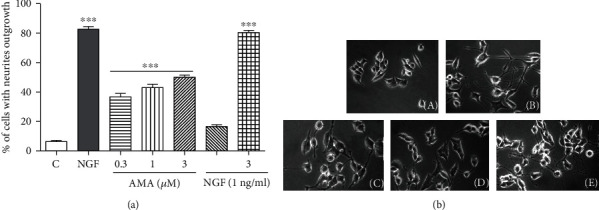
Neuritogenesis activity of amarogentin in PC12 cells. (a) Percentage of cells with neurite outgrowth after treatment with indicated doses of amarogentin and amarogentin with NGF for 48 h. (b) Morphological changes in the neurite outgrowth of PC12 cells treated with negative control (0.5% DMSO) (A), positive control (40 ng/ml NGF) (B), amarogentin (3 *μ*M) (C), NGF (1 ng/ml) (D), and amarogentin (3 *μ*M) with NGF (1 ng/ml) (E). Each experiment was repeated thrice. The data are presented as mean ± SEM. ^∗∗∗^ indicated significant and highly significant differences compared with the negative control group at *p* < 0.001.

The neuritogenic activity of amarogentin was evaluated in PC12 cells. Amarogentin induced neurite outgrowth in a dose-dependent manner. The percentage of cells with neurite outgrowth after treatment with 0, 0.3, 1, and 3 *μ*M amarogentin for 48 h was 6.3% ± 0.9%, 36.7% ± 2.3%, 43.0% ± 2.3%, and 50.0% ± 1.5%, respectively (Figure 7(a)). Interestingly, amarogentin (3 *μ*M) combined with NGF (1 ng/ml) remarkably increased the percentage of cells with neurite outgrowth from 50.0% ± 1.5% to 80.3% ± 1.5% (Figure 7(a)), and the neurite outgrowth interweaved into a network. The morphological changes in PC12 cells after treatment with DMSO, NGF (40 ng/ml), amarogentin (3 *μ*M), and amarogentin together with NGF are shown in Figure 7(b). The results suggest that amarogentin possessed NGF-mimic activity and could enhance NGF activity in PC12 cells. Amarogentin should be further studied given that it could be a candidate molecule because of its neuritogenic activity for treatment of neurodegenerative disorders.

## 4. Discussion

*G. rigescens* is a traditional Chinese herbal medicine which is used to treat inflammations, hepatitis, rheumatism, cholecystitis, and inflammation in China [16]. To search for the active components and understand the action mechanism of these compounds of *G. rigescens*, we began an intensive study on this species more than ten years ago. In our previous study, we used PC12 cells as a bioassay system to isolate 11 novel neuritogenic substances from *G. rigescens* and named as gentisides A–K [17, 18]. Furthermore, we used the fraction consisted with gentisides A-K to investigate the neuroprotection effects in AD model mice induced by scopolamine. We found that this fraction could improve the memory function of AD model mice in vivo [19]. In the present study, we focused on the isolation of antiaging molecules from *G. rigescens* by using a yeast replicative lifespan bioassay system. The chemical structure of amarogentin and changes in replicative lifespan of yeast in Figures 1(a) and 1(b) indicated that amarogentin has anantiaging effect on yeast.

Oxidative stress plays a crucial role in the aging process, and antioxidative stress mechanism is a strategy used to prevent and treat aging-related diseases, such as neurodegenerative diseases [11]. To understand the action mechanism of amarogentin for anti-aging, we firstly examined the effect of amarogentin on the survival rate of yeast under oxidative stress condition and activity of enzymes. The results in Figures 2(a) and 2(b) and the activities of total SOD and SOD2, CAT, and GPx (Figures 2(c)–2(g)) suggested that antioxidative stress and activity increase of enzymes are involved in antiaging effect of amarogentin. To obtain more evidences to support our conclusion, we investigated the gene expression of antioxidative enzyme and constructed *sod1*, *sod2*, *uth1*, and *skn7* mutants of yeast with K6001 background. We conducted replicative lifespan assays of *sod1*, *sod2*, *uth1*, and *skn7* mutants with a K6001 background; all of which had related effects on antioxidative stress. The results in Figures 3(a)–3(d) and Figures 4(a)–4(d) show that *SOD1*, *SOD2*, *CAT*, *GPX*, *UTH1*, and *SKN7* contribute to the antiaging effect of amarogentin. The results demonstrated that antioxidative stress is an important role for the antiaging effect of amarogentin.

In our research strategy, the yeast biological activity evaluation system has the advantages of low cost, short research period, and easy operation. This system was used for the initial screening and mechanism study. To confirm the effects of the antioxidative stress activity of amarogentin in higher organisms, we employed the PC12 cell line, which was derived from mammalian cells, as a bioassay system. A significant increase of the survival rates of PC12 cells under oxidative conditions as shown in Figure 5(b), significant reduction of ROS and MDA levels (Figures 5(c) and 5(d)), and increase of the total SOD and SOD2 activities (Figures 5(e)–5(g)) implied that amarogentin possessed the neuroprotective effect in PC12 cells via regulation of antioxidative stress. To understand which genes and signalling pathways were involved in antioxidative stress, we investigated both of the genes, *Nrf2* and *Bcl-x1* which are related to oxidative stress and neuroprotection [40, 41]. The increase of *SOD2*, *Nrf2*, and *Bcl-xl* gene expression after treatment of amarogentin (Figures 6(b)-6(d)) clarified that amarogentin produced neuroprotection via modification of antioxidative stress and regulation of *SOD2*, *Nrf2*, and *Bcl-xl* gene expression. These results were consistent with other reports [42].

The NGF is the first and best characterized neurotrophic factor [27]. NGF cannot pass through the blood brain barrier (BBB) because of its physical property, resulting in difficultly in using NGF as a drug to treat neurodegenerative diseases. Therefore, a small molecule that mimics or enhances the NGF activity and can pass through BBB can be a promising candidate for treatment of neurodegenerative diseases [43]. In the present study, we found that amarogentin not only significantly induced the neurite outgrowth but also enhanced the neuritogenic activity of NGF (Figures 7(a) and 7(b)). These results suggested that amarogentin has potential for treatment of neurodegenerative diseases, such as AD.

To confirm whether these obtained antiaging compounds have an anti-AD effect, PC12 cells were used to evaluate neuritogenic activity of them. Cucurbitacin B which is a triterpenoid and isolated from *Pedicellus melo*, like amarogentin, not only exerted antiaging effects on yeasts but also induced neurogenesis in PC12 cells and improved the memory of APP/PS1 transgenic mice [20, 44]. Amarogentin, which has a structure that is completely different to that of CuB, exhibits significant antiaging activity and NGF-mimic activity and enhances the NGF activity. The underlying mechanisms of the activities of amarogentin should be elucidated. Comparison of the similarity and difference of mechanisms of two different structures may be important for drug discovery.

## 5. Conclusion

Amarogentin isolated from *G. rigescens*, which is a TCM, showed a significant antiaging effect on yeasts and neuroprotective and neuritogenic activities in PC12 cells, a mammalian cell line. Amarogentin prolonged the replicative lifespan and neuroprotective activities by antioxidative stress activity. However, the underlying mechanisms of neuritogenic and antiaging activities need to be elucidated, and the relationship between these activities should also be addressed in future studies. Novel leading compounds can be designed and synthesised based on the structure of amarogentin. This study lays an important foundation for development of a novel drug for treatment of aging and neurodegenerative diseases.

## Data Availability

All figures and data used to support this study are included within this article.

## Supplementary Materials

Supplementary Materials The detailed method of measurement of SOD, GPx, and CAT enzymatic activities in yeast or PC12 cells; primer sequences used in this study; gene expression of *CAT*, and *GPx* in PC12 cells.
