# Supplementary material for: Amarogentin from Gentiana rigescens Franch Exhibits Antiaging and Neuroprotective Effects through Antioxidative Stress
Source: Oxid Med Cell Longev. 2020 Aug 1;2020:3184019. doi: 10.1155/2020/3184019 (PMC7421772; doi:10.1155/2020/3184019)
Supplement: Supplementary Materials — The detailed method of measurement of SOD, GPx, and CAT enzymatic activities in yeast or PC12 cells; primer sequences used in this study; gene expression of CAT, and GPx in PC12 cells. [file 3184019.f1.docx]

Materials and Methods

*Measurement of SOD, GPx and CAT Enzymatic Activity in Yeast or PC12 cells.*

For SOD enzyme activity assay, the 2 μg protein of each sample was taken, and SOD enzyme assay kit was used to determine the activity of superoxide enzyme. The process is as follows: At first, each sample was mixed with reagent 7, reacted for 1min to lost the sod 2 enzyme activity in the samples and got the supernatant after centrifugation as samples. The reagent I, blank control, samples, and the samples treated by reagent 7 were added to the 96 well plate, respectively. Afterwards, the reagent II, reagent III and reagent IV were added, mixed well and incubated at 37 ^o^C for 40 min. Final, the absorbance value of A550 of samples were measured after adding color reagent and standing at room temperature for 10 min. The activity of SOD enzyme was calculated according to the formula as following: [Control group OD value - Determination group OD value] / control group OD value / 50 × total volume of reaction solution / sample volume / protein concentration of sample to be measured.

For GPX enzyme activity assay, the 2 μg protein of each sample was taken and the total glutathione enzyme assay kit was used to determine glutathione enzyme activity. The general protocol is as follows: the glutathione peroxidase detection solution, samples, GPX detection working solution and peroxide reagent were added in 96 well plate in proper order. The absorbance value at A430 value every 5 minutes was measured for six times after mixing. The total glutathione activity was calculated according to the formula. The activity of GSH in the detection system was = [Δ A340 (sample) - Δ A340 (blank)] / (0.00622 × 0.276). Total glutathione activity in the sample = total glutathione activity in the detection system × dilution ratio / sample protein concentration.

For CAT enzyme activity assay, the 5 μg protein of each sample was taken and the catalase assay kit was used to measure catalase activity. The general operation is as follows: first, took different concentrations of hydrogen peroxide solution, added color working solution and reacted at 25 ^o^C for 15 minutes, then measured the absorption value of A250, and determined the standard curve of hydrogen peroxide concentration. Afterwards, added catalase buffer and 250 mM hydrogen peroxide in each sample. After reaction at 25 ^o^C for 1-5 minutes, added the enzyme reaction termination solution to terminate the reaction, then added the color working solution, reacted at 25 ^o^C for 15 minutes and measured the absorption value of A250. The concentration of hydrogen peroxide in the reaction system was calculated, and calculate the catalase activity was calculated as following. Sample catalase activity = [consumption of micromole of hydrogen peroxide] × [dilution ratio] / ([reaction minutes] × [sample volume] × [protein concentration]), [consumed micromole of hydrogen peroxide] = [blank control micromole of residual hydrogen peroxide] - [sample micromole of residual hydrogen peroxide]).


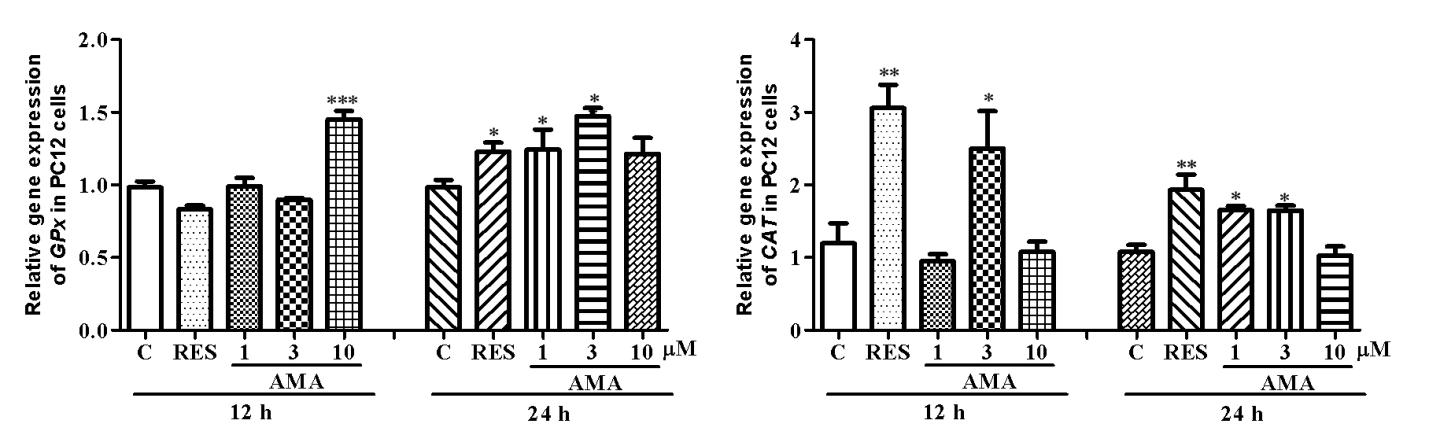


**Supplementary Figure 1** Effect of amarogentin on *Gpx* and *CAT* genes expression in PC12 cells after treatment of amarogentin and resveratrol for 12 h or 24 h. Experiments were repeated thrice, and the data were presented as means ± SEM. **p* < 0.05, ***p* < 0.01, ****p* < 0.001 compared with the control group.

**Supplementary Table 1** Primers sequences used in this study.

| Genes | Species | Sequences |
| --- | --- | --- |
| SOD1 | Yeast | Sense: 5′-CGC TCC GTC AAG TAA ACA TAG G-3′ |
|  | Yeast | Anti-sense: 5′-GGC CGC TGT TAT TGT TTT GAA C-3′ |
| SOD2 | Yeast | Sense: 5′-CTC CGG TCA AAT CAA CGA AT-3′ |
|  | Yeast | Anti-sense: 5′-CCT TGG CCA GAA GAT CTG AG-3′ |
| Gpx | Yeast | Sense: 5′-CGC TCC GTC AAG TAA ACA TAG G-3′ |
|  | Yeast | Anti-sense: 5′-GGC CGC TGT TAT TGT TTT GAA C-3′ |
| CAT | Yeast | Sense: 5′-TGA CAA ACT CCA CTG GTA ATC C-3′ |
|  | Yeast | Anti-sense: 5′-TCC CTG TTG AAA TGA GCC AA-3′ |
| TUB1 | Yeast | Sense: 5′-CCA AGG GCT ATT TAC GTG GA-3′ |
|  | Yeast | Anti-sense: 5′-GGT GTA ATG GCC TCT TGC AT-3′ |
| SOD1 | Rat | Sense: 5'-AGG GCA TCA TCA ATT TCG AGC-3’ |
|  | Rat | Anti-sense: 5′-ACA TTG CCC AAG TCT CCA AC-3’ |
| SOD2 | Rat | Sense: 5'-GGA AGC CAT CAA ACG TGA CT-3′ |
|  | Rat | Anti-sense: 5′-CCT TGC AGT GGA TCC TGA TT-3′ |
| Gpx | Rat | Sense: 5′-GAC ATC AGG AGA ATG GCA AGA-3′ |
|  | Rat | Anti-sense: 5′-CAC CTC GCA CTT CTC AAA CA-3′ |
| CAT | Rat | Sense: 5'-TCA GCG TTT GGT GGA GAA-3′ |
|  | Rat | Anti-sense: 5′-GCC TGG CTC ATC TTT ATC-3′ |
| Bcl-x1 | Rat | Sense: 5'-TTC GGG ATG GAG TAA ACT GG-3' |
|  | Rat | Anti-sense: 5'-TGT CTG GTC ACT TCC GAC TG-3' |
| Nrf2 | Rat | Sense: 5'-TGG TGG TTT GCT ACG ACG-3′ |
|  | Rat | Anti-sense: 5′-CTC CAG AAC TCC AGG CGG-3′ |
| GAPDH | Rat | Sense: 5'-CAG CCT CGT CTC ATA GAC AAG ATG-3' |
|  | Rat | Anti-sense: 5′-CAA TGT CCA ACT TTG TCA CAA GAG AAA-3' |
